# AFF4 Loss Compromises Osteogenesis of Orofacial Mesenchymal Stem Cells During Alveolar Bone Repair via AMPK‐Mediated Mitophagy

**DOI:** 10.1111/cpr.70284

**Published:** 2026-09-29

**Authors:** Li Zhu, Shuo Chen, Guowen Luo, Tingyue Zhang, Hengyi Lin, Yiling Chen, Chenchen Zhou, Shujuan Zou

**Affiliations:** ^1^ State Key Laboratory of Oral Diseases, National Clinical Research Center for Oral Diseases, West China Hospital of Stomatology Sichuan University Chengdu China; ^2^ Department of Pediatric Dentistry, West China Hospital of Stomatology Sichuan University Chengdu China; ^3^ Department of Orthodontics, West China Hospital of Stomatology Sichuan University Chengdu China

**Keywords:** AFF4, alveolar bone repair, AMPK signalling, mitophagy, orofacial mesenchymal stem cells (OMSCs), osteogenic differentiation

## Abstract

Repair of maxillofacial bone defects presents significant clinical challenges. Orofacial mesenchymal stem cells (OMSCs) mediate maxillofacial bone repair, while the molecular mechanisms governing osteogenic commitment of OMSCs remain incompletely understood. Here, we investigate the function of AF4/FMR2 family member 4 (AFF4) in OMSCs osteogenesis and maxillofacial bone healing. By using a tamoxifen‐inducible *Aff4* knockout mouse model, we demonstrate that AFF4 depletion significantly delays the alveolar bone repair post‐tooth extraction. OMSCs of *Aff4* knockout mice exhibit decreased expression of osteogenic markers (RUNX2, ALPL, OSX, COL1A1) and reduced mineralization in vitro. Mechanistically, AFF4 loss suppresses AMPK signalling and downstream mitophagy, manifested by reduced Parkin translocation and LC3B recruitment to mitochondria, and impaired mitophagosome‐lysosome fusion. Notably, the rescue of mitophagy and the osteogenic capacity of *Aff4*‐deficient OMSCs by AICAR (an AMPK activator) was abolished by Compound C (an AMPK inhibitor). Collectively, our findings establish the critical role of AFF4 in maxillofacial bone regeneration and demonstrate that it functions through the AMPK‐mitophagy axis in OMSCs. This study provides a novel insight into the function of AFF4 in maxillofacial bone biology.

## Graphical abstract

Compromised alveolar socket healing following tooth extraction upon *Aff4* knockout is caused by suppressed osteogenic capacity of orofacial mesenchymal stem cells, which results from defective AMPK‐mediated mitophagy.

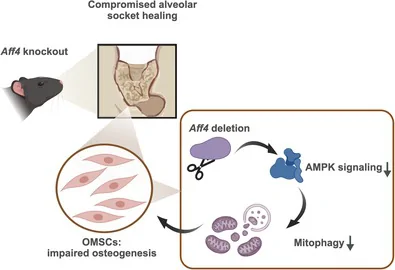

## 1 Introduction

Maxillofacial bones are essential for mastication, speech and facial aesthetics [1]. Maxillofacial bone defects, caused by trauma, inflammation, or congenital anomalies, compromise patients' quality of life and impose challenges to clinical management [2]. Notably, maxillofacial bones differ from other skeletal bones in terms of embryonic origin and homeostatic mechanisms [1]. Orofacial mesenchymal stem cells (OMSCs), inherent stem cells residing in jaw bones, originate from ectodermal neural crest cells [3]. Given the intrinsic and niche‐specific variations, OMSCs exhibit unique properties compared to mesenchymal stem cells (MSCs) of long bones. Specifically, OMSCs exhibit superior proliferative, osteogenic and angiogenic potential [3, 4]. OMSCs are particularly promising for craniomaxillofacial defect repair due to their easier accessibility, reduced trauma and lower immunogenicity [5, 6]. Therefore, in‐depth exploration of the molecular regulatory mechanisms governing osteogenic differentiation of OMSCs is critical for developing novel therapeutic strategies for maxillofacial bone defect regeneration.

AF4/FMR2 family member 4 (AFF4) works as the scaffold to assemble and stabilize super elongation complex (SEC) [7]. AFF4/SEC enables rapid transcriptional induction by releasing paused RNA polymerase II from the promoter‐proximal site [8]. Beyond its established roles in tumorigenesis [7, 9, 10] and HIV transactivation [11, 12, 13, 14], AFF4 has also been found to be implicated in bone biology over the past decade. Germline AFF4 missense mutations cause CHOPS syndrome, a developmental disorder in which skeletal involvement is universally observed [15, 16]. Additionally, AFF4 has been shown to regulate osteogenic commitment of several kinds of stem cells, including human MSCs, dental follicle cells and periodontal ligament stem cells (PDLSCs) [17, 18]. Nevertheless, the role of AFF4 in OMSCs‐mediated maxillofacial bone repair remains elusive.

Mitophagy is an organelle‐specific autophagic process where autophagosomes engulf damaged mitochondria and then fuse with lysosomes for degradation. In canonical Parkin‐dependent mitophagy, E3 ubiquitin ligase Parkin translocates from the cytosol to mitochondria and is activated by PINK1 on the outer mitochondrial membrane. Activated Parkin ubiquitinates outer mitochondrial membrane proteins, which then recruit core autophagy machinery, ultimately leading to autophagosome formation and mitophagy initiation [19]. AMP‐activated protein kinase (AMPK), a known autophagy regulator [20], has been shown to govern the early stage of Parkin‐mediated mitophagy. Specifically, AMPK phosphorylates Parkin on Ser108 through both ULK1‐dependent and ULK1‐independent mechanisms, causing its translocation to mitochondria and subsequent full activation by PINK1 [21, 22, 23]. A growing number of studies suggest AMPK signalling drives osteogenesis via mitophagy [24, 25]. Emerging evidence indicates that AFF4 regulates AMPK expression. Komori et al. reported that AFF4 regulates AMPKα2 transcription in the mouse hypothalamic neuronal cell line GT1‐7. Using luciferase assays, they demonstrated that AFF4 overexpression significantly increased AMPKα2 promoter activity compared with mock‐transfected controls [26]. Moreover, RNA‐seq analysis of human PDLSCs revealed downregulation of AMPKα2 and significant enrichment of mitophagy‐related pathways following siRNA‐mediated AFF4 knockdown [18]. Hence, we speculate that AFF4 functions during maxillofacial bone regeneration by regulating AMPK‐mediated mitophagy.

To test our hypothesis, we generated a tamoxifen‐inducible *Aff4* knockout mouse model to examine how AFF4 influences alveolar bone healing and the osteogenic potential of OMSCs. Our experiments demonstrate that AFF4 loss leads to compromised osteogenesis and defective alveolar bone repair. Mechanistically, AFF4 depletion suppresses the osteogenic capability of OMSCs, partially through the modulation of AMPK‐mediated mitophagy.

## 2 Materials and Methods

### 2.1 Mice Breeding and Surgery

*Aff4* ^*fl/fl*^ mice, with loxP sites inserted to flank exons 2 and 3 of the *Aff4* gene, were produced by Biocytogen Pharmaceuticals (Beijing, China). We crossed *CAG‐iCre* male mice (GemPharmatech, Nanjing, China) and *Aff4* ^*fl/fl*^ female mice to obtain *CAG‐iCre*; *Aff4* ^*fl/fl*^ mice. Global *Aff4* knockout was induced in 3‐week‐old *CAG‐iCre*; *Aff4* ^*fl/fl*^ mice by daily intraperitoneal injection of tamoxifen (0.15 mg/g body weight; J60955.06; ThermoFisher) for three consecutive days. *Aff4* ^*fl/fl*^ littermates injected with tamoxifen served as controls. One week after injection, an alveolar defect repair model was established by extracting the maxillary right first molar in *CAG‐iCre*; *Aff4* ^*fl/fl*^ and *Aff4* ^*fl/fl*^ male mice. Maxillae were harvested at 7, 14 and 21 days post‐tooth extraction for subsequent analysis. Animal experiments were conducted with ethical approval (WCHSIRB‐AT‐2025‐543).

### 2.2 Microcomputed Tomography (Micro‐CT) Analysis

Jaws from *CAG‐iCre*; *Aff4* ^*fl/fl*^ mice and *Aff4* ^*fl/fl*^ littermates were fixed with 4% paraformaldehyde for 24–48 h and kept in PBS until scanning. Scanning was performed with Venus Micro CT (PingSeng Scientific, China), followed by three‐dimensional volumetric reconstruction. The palatal root socket of the extracted first molar was defined as the region of interest. Thresholds were set to encompass all trabecular and cortical bone and were maintained consistently across all groups.

### 2.3 Histological Staining

The samples were processed into paraffin sections after fixation, decalcification and dehydration. The sections were deparaffinized and rehydrated before staining. Haematoxylin and Eosin (H&E), Alkaline phosphatase (ALP) and tartrate‐resistant acid phosphatase (TRAP) staining were performed using commercial kits (H&E: BL735A, Biosharp; TRAP/ALP: 294‐67001, Wako) following the manufacturer's instructions.

For immunohistochemical (IHC) staining, VECTASTAIN ABC‐HRP Kit (PK‐4001, Vector Laboratories) and DAB peroxidase substrate kit (SK‐4100, Vector Laboratories) were utilized. Briefly, sections were immersed in sodium citrate buffer (P0083, Beyotime) at 100°C for 20 min for antigen retrieval, followed by incubation with 3% H_2_O_2_ (AR1108, Boster) for 30 min for endogenous peroxidase inhibition. Afterwards, samples were incubated with anti‐OSX antibody (HA722817, Huabio) overnight at 4°C, and then with biotinylated secondary antibody for 2 h at room temperature. Subsequently, the DAB kit was used to perform immunolabelling and the nuclei were stained with haematoxylin. Section images were captured with Tissue Slide Scanner VS120 (Olympus). OSX‐positive cells and TRAP‐positive cells within the extraction sockets were counted and quantified using FIJI software (ImageJ, National Institutes of Health).

### 2.4 OMSCs Isolation and Osteogenic Induction

OMSCs were extracted and cultured as previously reported [4, 27, 28]. Briefly, mandibles of 4‐week‐old male mice were dissected, cut into small pieces and digested enzymatically at 37°C for 1 h with collagenase I (3 mg/mL; BioFroxx) and dispase II (4 mg/mL; Solarbio). After passing through a 70‐μm cell strainer, the cells were centrifuged and resuspended in DMEM (Gibco) with 10% foetal bovine serum (FBS; Gibco) and 1% penicillin–streptomycin (Gibco). Cells were cultured in a humidified incubator (37°C, 5% CO_2_) with medium renewed every 2 days. Cells were passaged upon reaching 80% confluence to obtain passages 2–5 for subsequent experiments.

To evaluate the proliferative capacity of OMSCs, the CCK‐8 kit (K1018, APExBIO) was utilized and absorbance at 405 nm was detected by SpectraMax iD3.

l‐ascorbic acid (50 μg/mL; Psaitong), dexamethasone (10 nM; Solarbio) and β‐glycerolphosphate (8 mM; Psaitong) were added into culture medium for osteogenic induction. 5‐aminoimidazole‐4‐carboxamide ribonucleotide (AICAR; 100 μM; S1515, Beyotime) and Compound C (CC; 10 μM; B3252, APExBIO) were added to activate or inhibit AMPK signalling, respectively.

### 2.5 Protein Extraction and Western Blot (WB)

For WB analysis of maxillary bone tissue, total proteins were extracted from mice at 7 days post‐tamoxifen administration [29], and at 7 and 14 days post‐surgery. For WB analysis of OMSCs, total cellular proteins were obtained after osteogenic induction for 5 days.

RIPA buffer (P0013B, Beyotime) supplemented with 1% protease inhibitor cocktail (P1005, Beyotime) was used to extract proteins. Following SDS‐PAGE separation, proteins were electroblotted onto PVDF membranes (Millipore). After blocking for 2 h with bovine serum albumin (BSA, 5%, BioFroxx), the blots were incubated with primary antibodies (overnight, 4°C), and then with HRP‐conjugated secondary antibodies (2 h, room temperature). Bands detection was performed with ECL reagent (K1231, APExBIO) on a ChemiDoc system (Bio‐Rad). The optical density of each protein band was quantified using FIJI software and normalized to the corresponding ACTB loading controls. Primary and secondary antibodies used in this experiment were provided in Table S1.

### 2.6 RNA Extraction and Quantitative Real‐Time Polymerase Chain Reaction (qRT‐PCR)

Total RNA was isolated from OMSCs using TRIzol (Invitrogen), and its concentration was measured with a NanoDrop OneC spectrophotometer (Thermo Fisher Scientific). Reverse transcription was conducted using the PrimeScript FAST RT reagent kit with gDNA Eraser (RR092A; TaKaRa). Subsequently, qRT‐PCR was performed with TB Green Premix *Ex Taq* II FAST qPCR (CN830A; TaKaRa) on a Roche LightCycler 480 II system (Switzerland). The 2^−ΔΔ*Ct*^ method was used to calculate relative mRNA levels of targeted genes, with *Gapdh* as the endogenous reference. The primers used were listed in Table S2.

### 2.7 Alkaline Phosphatase (ALP) Staining and Alizarin Red Staining

Cells were cultured in 48‐well plates and subjected to osteogenic induction. 4% paraformaldehyde was used for cell fixation for 15 min before staining. BCIP/NBT staining kit (C3206, Beyotime) was utilized for ALP staining after 7 days of induction. 1% Alizarin Red S solution (G1452, Solarbio) was used to stain calcified nodules after 14 days of osteogenic induction.

### 2.8 Immunofluorescence (IF) Staining

Cells were first treated with MitoTracker Red CMXRos (200 nM; M7512, Thermo Fisher Scientific) for 15 min to label mitochondria. Subsequently, the cells were fixed with paraformaldehyde (4%, 10 min), permeabilized with Triton X‐100 (0.25%, 10 min) and blocked with BSA (5%, 1 h). Immunolabelling was performed by incubation with primary antibodies (4°C, overnight) and then with secondary antibody (room temperature, 2 h). Antibodies used in this experiment included anti‐LC3B (R382687, ZENBIO), anti‐Parkin (HA722952, Huabio) and secondary antibody (Alexa Fluor 488; A‐11008, Thermo Fisher Scientific). Nuclei were counterstained with DAPI (C0065, Solarbio). Immunofluorescent images were captured with SpinSR microscope (Olympus).

### 2.9 Transmission Electron Microscopy

After mitophagy induction with carbonyl cyanide m‐chlorophenyl hydrazone (CCCP; 10 μM; HY‐100941, MedChemExpress) for 2 h, cells were harvested by trypsinization and centrifugation. Cell fixation was performed by initial fixation with 2.5% glutaraldehyde and secondary fixation with 1% osmium tetroxide. Mitophagy was detected using a transmission electron microscope (JEM‐1400FLASH, JEOL).

### 2.10 Mitochondrial Analysis

Cells were seeded in confocal dishes and cultured either with or without AICAR treatment. The Enhanced Mitochondrial Membrane Potential Assay Kit with JC‐1 (C2003S, Beyotime), MitoSOX Red (M36008, Invitrogen) and Reactive Oxygen Species Assay Kit (S0033S, Beyotime) were used as the manufacturer instructed. A combination of MitoTracker Green (C1048, Beyotime) and LysoTracker Red (C1046, Beyotime) was used to visualize mitochondria and lysosomes. After removal of residual fluorescence probes, the dishes were mounted and observed under a live cell imaging system (Olympus). For MitoSOX and cellular ROS staining, the mean fluorescence intensity was measured using FIJI software. For JC‐1 staining, the red/green fluorescence intensity ratio was calculated to evaluate mitochondrial membrane potential.

### 2.11 Statistical Analysis

All mice (*n* = 5 per group) underwent the same tooth extraction surgery. Micro‐CT and histological data were acquired and quantified by investigators blinded to group allocation. Micro‐CT analysis was performed with five mice per group, and histological staining was based on three randomly selected samples per group. All other experiments were performed with three independent biological replicates. All quantitative results are displayed as mean ± standard deviation (SD). Comparison between two groups was performed using Student's *t*‐test. One‐way ANOVA with Tukey's multiple comparisons test was applied for comparisons among multiple groups. A *p*‐value of less than 0.05 was defined as statistically significant. GraphPad Prism 10.0 was used for all statistical analyses.

## 3 Results

### 3.1 *Aff4* Loss Leads to Delayed Alveolar Socket Healing

Firstly, an alveolar bone repair model was established, and the expression pattern of AFF4 during the healing process in wild‐type mice was examined. WB analysis of maxillary bone tissue revealed that AFF4 protein levels were significantly higher at Days 7 and 14 than at Day 0, with a more prominent increase at Day 7 (Figure 1A). To clarify the role of AFF4 in alveolar bone repair, 3‐week‐old *CAG‐iCre*; *Aff4* ^*fl/fl*^ (hereafter, KO) mice and *Aff4* ^*fl/fl*^ (hereafter, WT) littermates were subjected to tamoxifen administration and subsequent tooth extraction procedure (Figure 1B). One week after tamoxifen induction, WB assays confirmed effective knockout of AFF4 (Figure S1A). Three weeks post‐induction, KO mice showed similar body size to the WT littermates (Figure S1B), and no significant difference in the gross morphology of the jaw bones was observed (Figure S1C–E). Micro‐CT analysis showed delayed alveolar bone healing of KO mice at all evaluated time points. At Day 21, the extraction sockets of WT mice were nearly filled with new bone, while KO mice showed reduced bone fill and a more irregular bone surface (Figure 1C–E).

**FIGURE 1:**
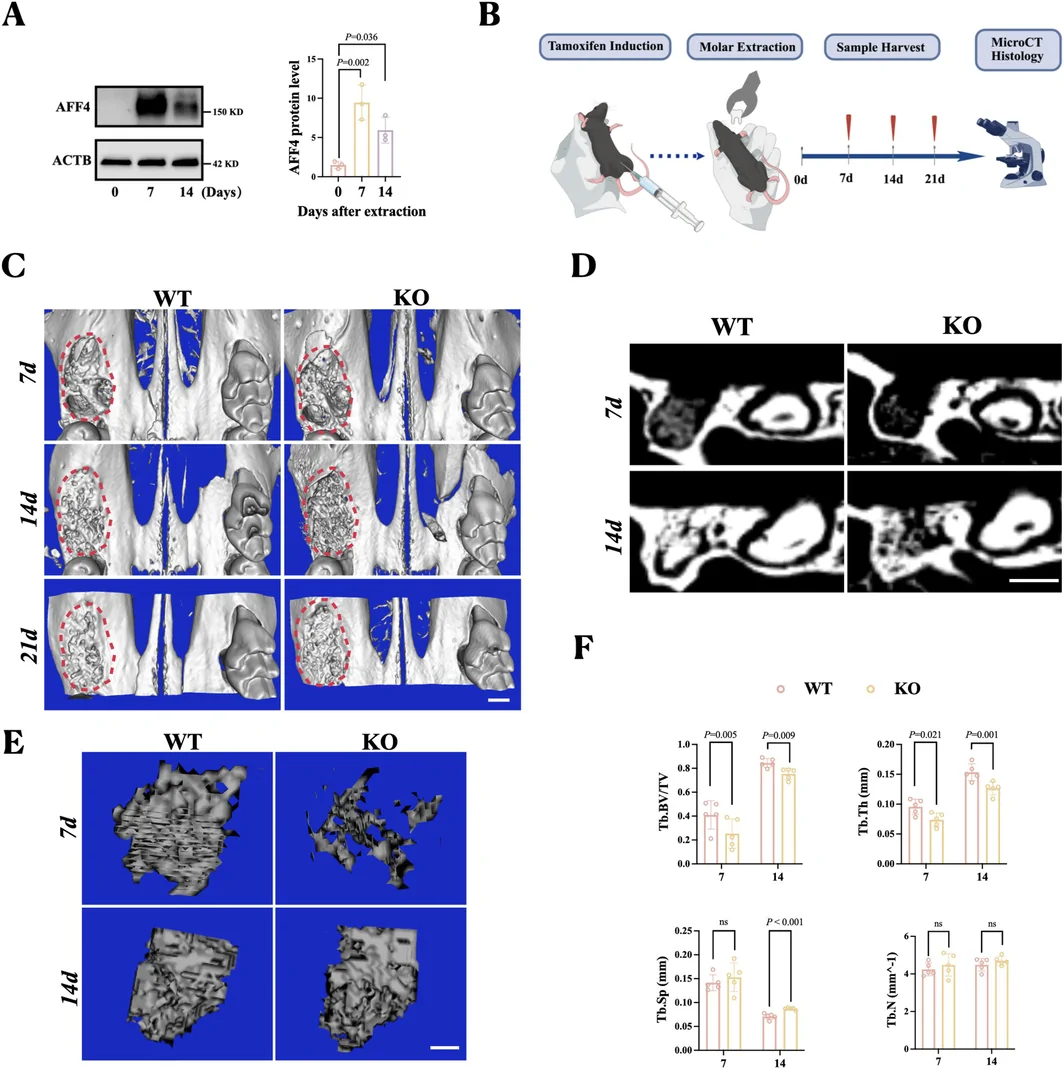
*Aff4* knockout mice exhibit compromised extraction socket healing. (A) Western blot (WB) assay for AFF4 protein expression during extraction socket healing (left panel) and quantified levels normalized to ACTB (right panel) (*n* = 3). (B) Flowchart of the animal experiment study design (Figure created with BioGDP.com). Three‐week‐old mice were treated with tamoxifen for 3 consecutive days to induce *Aff4* knockout (KO). Maxillary molar extraction was performed 1 week after induction (Day 0), and the mice were sacrificed on Days 7, 14 and 21 to collect maxillae for subsequent analysis. (C) Representative images of three‐dimensional micro‐CT reconstructions of maxillae (occlusal view) at Days 7, 14 and 21. Red dashed lines show the extraction socket. Scale bar: 500 μm. (D) Representative images of micro‐CT sagittal sections of maxillae on Days 7 and 14. Scale bar: 500 μm. (E) Representative images of three‐dimensional micro‐CT reconstructions of bone mass in the extraction socket. Scale bar: 200 μm. (F) Quantitative micro‐CT analysis of new bone mass at Days 7 and 14 (*n* = 5). BV/TV, bone volume fraction; Tb.N, trabecular number; Tb.Th, trabecular thickness; Tb.Sp, trabecular separation. KO, *CAG‐iCre*; *Aff4* ^*fl/fl*^; WT, *Aff4* ^*fl/fl*^.

Due to the difficulty in delineating the extraction socket boundaries at Day 21, only samples collected at Days 7 and 14 were employed for subsequent analyses. Quantitative analysis of micro‐CT data demonstrated that at Days 7 and 14, bone volume fraction (BV/TV) and trabecular thickness (Tb.Th) of newly formed bone in extraction sockets were lower in KO mice than in WT mice, whereas trabecular number (Tb.N) and trabecular separation (Tb.Sp) did not differ significantly between the two groups (Figure 1F).

Histological techniques were further employed to characterize socket healing at Days 7 and 14. Consistent with micro‐CT findings, Haematoxylin and Eosin (H&E) staining showed that bone newly formed in extraction sockets of KO mice was reduced compared to WT mice at Days 7 and 14 (Figure 2A). We further investigated osteogenic and osteoclastic activity during alveolar socket healing. Alkaline phosphatase (ALP) staining demonstrated that alkaline phosphatase activity in WT mice remained consistently high at Days 7 and 14. In contrast, KO mice exhibited significantly lower ALP activity at Day 7. Although ALP activity in KO mice increased slightly at Day 14 compared to Day 7, it remained lower than that of WT mice at both time points (Figure 2B). Immunohistochemistry staining showed that cells expressing OSX, the osteoblastic marker, were markedly more abundant in WT mice than in KO mice (Figure 2C,D). Tartrate‐resistant acid phosphatase (TRAP) staining, however, detected no significant group difference in osteoclastic activity on Day 7 or Day 14 (Figure 2E,F).

**FIGURE 2:**
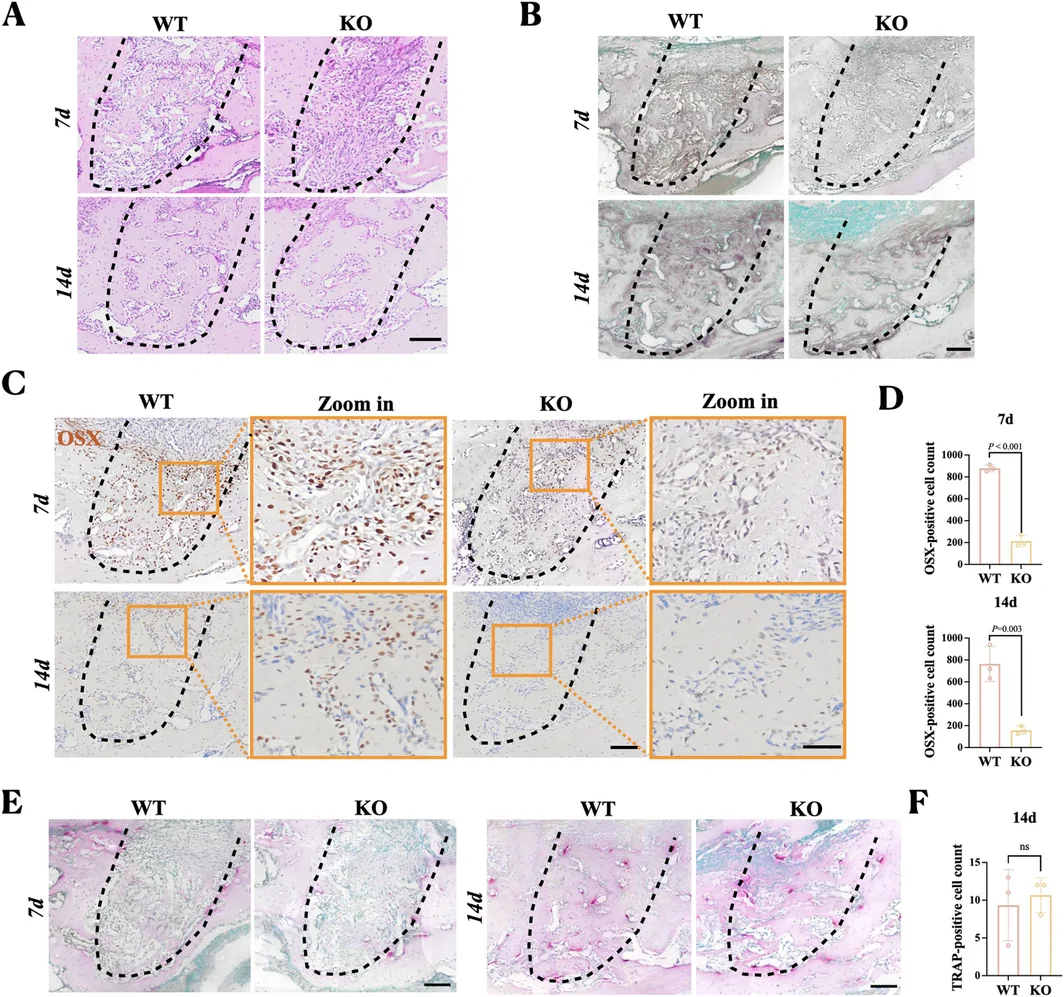
Effects of AFF4 ablation on osteogenic and osteoclastic activity in extraction sockets. (A) Representative images of haematoxylin and eosin staining of extraction sockets at Days 7 and 14. Scale bar: 100 μm. (B) Representative alkaline phosphatase (ALP) staining images of extraction sockets at Days 7 and 14. Scale bar: 100 μm. (C) Representative immunohistochemical staining images of OSX‐positive cells in extraction sockets at Days 7 and 14. Scale bars: 100 μm (low magnification); 50 μm (high magnification). (D) Histograms show OSX‐positive cell count in extraction sockets (*n* = 3). (E) Representative images of tartrate‐resistant acid phosphatase (TRAP) staining of extraction sockets at Days 7 and 14. Scale bar: 100 μm. (F) Histogram shows quantification of TRAP‐positive cells in extraction sockets at Day 14 (*n* = 3). The extraction socket boundary in (A, B, C, E) was traced by the yellow dashed line.

Taken together, AFF4 expression is elevated during extraction socket healing and inducible *Aff4* knockout compromises osteogenic activity and alveolar bone repair, whereas osteoclastic activity remains unaffected at the early healing stage.

### 3.2 *Aff4* Ablation Compromises Osteogenic Differentiation of OMSCs

Considering OMSCs are the key drivers of maxillofacial defect healing [30], we further investigated whether AFF4 ablation affects osteogenic differentiation of these niche‐specific stem cells. OMSCs of WT and KO mice were isolated from mandibles via enzyme digestion (Figure 3A). Those OMSCs expressed CD44 and CD90, the MSC surface markers (Figure S2A,B). AFF4 deletion was confirmed by significantly reduced mRNA and protein levels using qRT‐PCR and WB (Figure 3B,C). Cell proliferation capacity was not significantly altered by AFF4 deficiency, as determined by CCK‐8 assays (Figure 3D). After osteogenic induction, OMSCs of KO mice expressed fewer osteogenic markers, including RUNX2, ALPL, OSX and COL1A1 (Figure 3E,F). Besides, ALP activity and calcium nodule deposition in KO OMSCs were also reduced compared to those of WT OMSCs (Figure 3G,H). Overall, these observations reveal that loss of *Aff4* inhibits the osteogenic commitment of mouse OMSCs.

**FIGURE 3:**
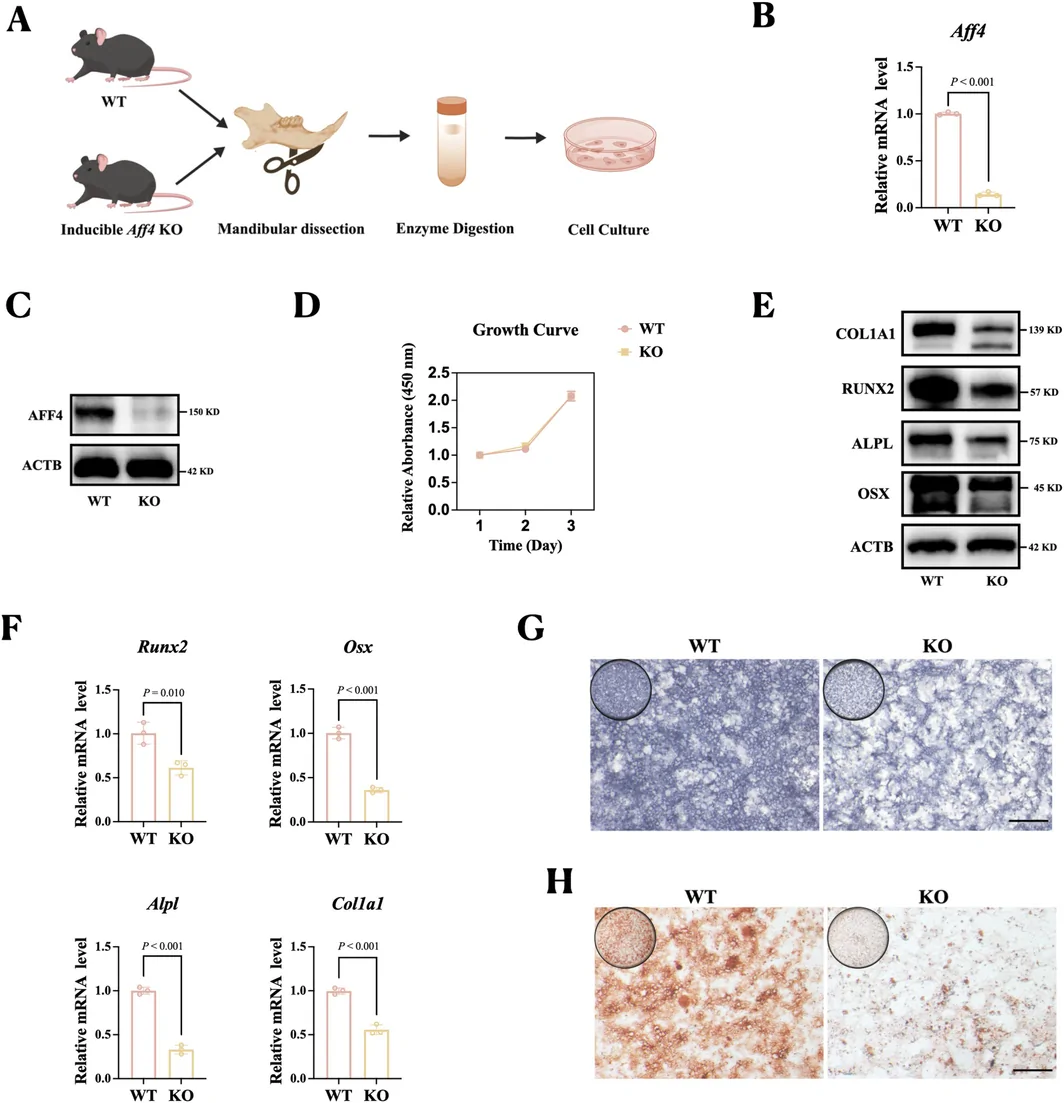
Osteogenic potential of OMSCs is inhibited upon AFF4 deletion. (A) Schematic diagram of extracting orofacial mesenchymal stem cells (OMSCs) from mouse mandibles (Figure created with BioGDP.com). (B) Detection of *Aff4* mRNA transcripts in OMSCs by quantitative real‐time polymerase chain reaction (qRT‐PCR) assays. (C) AFF4 protein levels in OMSCs measured by WB analysis. (D) Growth curves of OMSCs. Data are presented as fold change of CCK‐8 assay absorbance (450 nm) relative to Day 1. (E) WB analysis showing the protein expressions of COL1A1, RUNX2, ALPL and OSX in OMSCs after 5 days of osteogenic induction. (F) The mRNA levels of *Runx2*, *Osx*, *Alpl* and *Col1a1* in OMSCs measured by qRT‐PCR after 5 days of osteogenic induction. (G) Representative ALP staining image of OMSCs after 7 days of osteogenic induction. Scale bar, 500 μm. (H) Representative Alizarin Red S (ARS) staining images of OMSCs after 14 days of osteogenic induction. Scale bar, 500 μm.

### 3.3 *Aff4* Knockout Inhibits Mitophagy in OMSCs

AMPK is a heterotrimeric complex in which the α subunit serves as the catalytic component. In mammals, two α‐isoforms, α1 and α2, are encoded by different genes with disparate tissue distribution [31]. Therefore, the expression profile of α isoforms of AMPK in OMSCs was first assessed. qRT‐PCR showed that α1 was the dominant isoform, which was down‐expressed upon AFF4 deletion in OMSCs (Figure 4A). Thr172 phosphorylation in the kinase domain of the α subunit causes significant functional activation of AMPK and is commonly used as a marker of its activated form [32]. WB analysis showed that both p‐AMPK (Thr172) and total AMPK protein levels were decreased in *Aff4*‐KO OMSCs, whereas the expression of PINK1 and Parkin remained unchanged compared to WT cells (Figure 4B). *Aff4*‐KO OMSCs also exhibited reduced LC3B‐II level alongside elevated TOMM20 and SQSTM1/p62. Following CQ treatment to block lysosomal degradation, the LC3‐II/I ratio increased in WT cells, accompanied by TOMM20 accumulation; however, in *Aff4*‐KO cells, the LC3‐II/I ratio showed only a modest increase without a concomitant change in TOMM20 (Figure 4B). These findings confirm that AFF4 deficiency suppresses both basal mitophagy and mitophagic flux in OMSCs.

**FIGURE 4:**
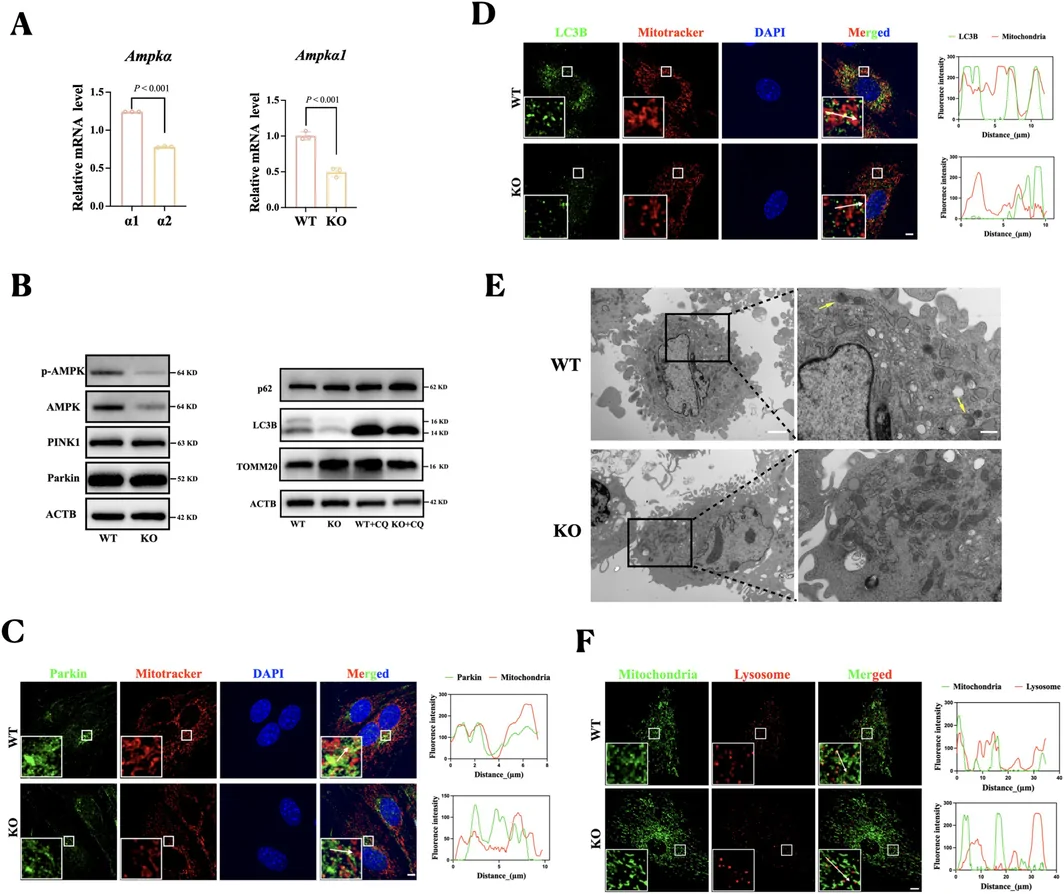
AFF4 deficiency blocks mitophagy in OMSCs. (A) qRT‐PCR measuring *Ampk* expression in OMSCs: *Ampkα1* and *Ampkα2* in WT OMSCs (left panel), and *Ampkα1* in WT and KO OMSCs (right panel). (B) Protein levels of p‐AMPK, AMPK, PINK1, Parkin, p62, TOMM20 and LC3B in OMSCs assessed by WB analysis. Chloroquine (CQ, 40 μM, 2 h). (C, D) Immunofluorescent (IF) staining images showing colocalization of mitochondria (MitoTracker Red CMXRos, red) with Parkin (green) or LC3B (green) in OMSCs. Nuclei were stained by DAPI (blue). Intensity profiles on the right panel represent the colocalization of Parkin (green) or LC3B (green) with mitochondria (red) along the indicated lines. Scale bar: 5 μm. (E) Representative transmission electron microscopy images of OMSCs. Mitochondria sequestered by a double‐membraned vesicle are indicated using yellow arrows. Scale bar: 2 μm (left panel), 500 nm (right panel). (F) Live cell images showing colocalization of mitochondria (MitoTracker, green) with lysosome (LysoTracker, red) in OMSCs. Intensity profiles on the right depict the colocalization of mitochondrial (green) and lysosomal (red) fluorescence signals along the indicated lines. Scale bar: 10 μm.

Previous studies have revealed that Parkin translocation is regulated by AMPK [21, 22, 23]. Given that *Aff4* loss downregulates AMPK expression without altering PINK1 and Parkin, we next examined whether *Aff4* deficiency affects Parkin mitochondrial recruitment by immunofluorescence co‐localization. IF staining showed that *Aff4*‐KO OMSCs exhibited less overlapping fluorescent signals between Parkin and mitochondria (Figure 4C). Meanwhile, reduced LC3B fluorescent puncta and decreased colocalization of LC3B with mitochondria were evident in *Aff4*‐KO OMSCs compared to WT cells (Figure 4D). Although autophagosomes (bilayer membrane‐bounded vesicles) were detected in both WT and KO OMSCs by transmission electron microscopy upon CCCP induction, mitophagosomes (mitochondria containing autophagosomes) were barely observed in KO OMSCs (Figure 4E). Mitophagy culminates with mitophagosome‐lysosome fusion and final degradation [33]. The fusion event was visualized by live‐cell imaging using LysoTracker Red and MitoTracker Green. Confocal microscopy demonstrated that fewer merged (yellow) signals were detected in *Aff4*‐KO OMSCs (Figure 4F).

Given the essential role of mitophagy in mitochondrial homeostasis, we also assessed the effect of AFF4 deficiency on mitochondrial status. Confocal microscopy revealed that *Aff4*‐KO OMSCs, in contrast to WT OMSCs, displayed extensive mitochondrial fragmentation and marked collapse of mitochondrial membrane potential (Figure S2C–E). Notably, both mitochondrial superoxide and cellular ROS showed significant accumulation in *Aff4*‐KO OMSCs, suggesting that defective mitophagy compromises the clearance of dysfunctional mitochondria, which further leads to gross ROS accumulation and subsequent cellular dysfunction (Figure S2F–I). Mitochondrial fusion and fission, as integral components of mitochondrial homeostasis, are closely linked to mitophagy [34]. We therefore examined the protein levels of key mitochondrial fusion and fission markers and found no significant differences between *Aff4*‐KO and WT OMSCs (Figure S2J). Collectively, these data indicate that defective mitophagy in *Aff4*‐KO OMSCs is attributable to failure to recruit the autophagic machinery, rather than to aberrant mitochondrial dynamics.

Taken together, our findings support that AFF4 loss inhibits AMPK expression and mitophagy in OMSCs. Mitophagy inhibition is evidenced by decreased Parkin translocation, as well as reduced co‐localization of both LC3B and lysosomes with mitochondria.

### 3.4 AMPK Activation Rescues Mitophagy and Osteogenesis of *Aff4* Knockout OMSCs

To determine whether AMPK mediates the effect of AFF4 on mitophagy and osteogenesis, AMPK agonist AICAR was first applied to reactivate AMPK signalling in *Aff4*‐KO OMSCs. IF assays showed that AICAR treatment promoted the mitochondrial localization of both Parkin and LC3B in *Aff4*‐KO OMSCs (Figure 5A,B). Consistently, AICAR upregulated the mRNA and protein levels of RUNX2, ALPL, OSX and COL1A1 (Figure 5C,D). Meanwhile, WB assays further revealed increased p‐AMPK (Thr172) and LC3B‐II/I ratio, along with decreased p62 level upon AICAR stimulation (Figure 5D). ALP and ARS staining suggested that AICAR treatment increased ALP activity and calcium nodule formation in *Aff4*‐KO OMSCs (Figure 5E,F). To verify that these effects were specifically mediated by AMPK, we treated *Aff4*‐KO OMSCs with the AMPK inhibitor Compound C to suppress AICAR‐induced AMPK activation. As expected, Compound C effectively abrogated AICAR‐induced rescue of mitophagy and osteogenic differentiation in *Aff4*‐KO OMSCs, as shown by WB analysis (Figure 5G). This was further corroborated by ALP and ARS staining (Figure 5H,I).

**FIGURE 5:**
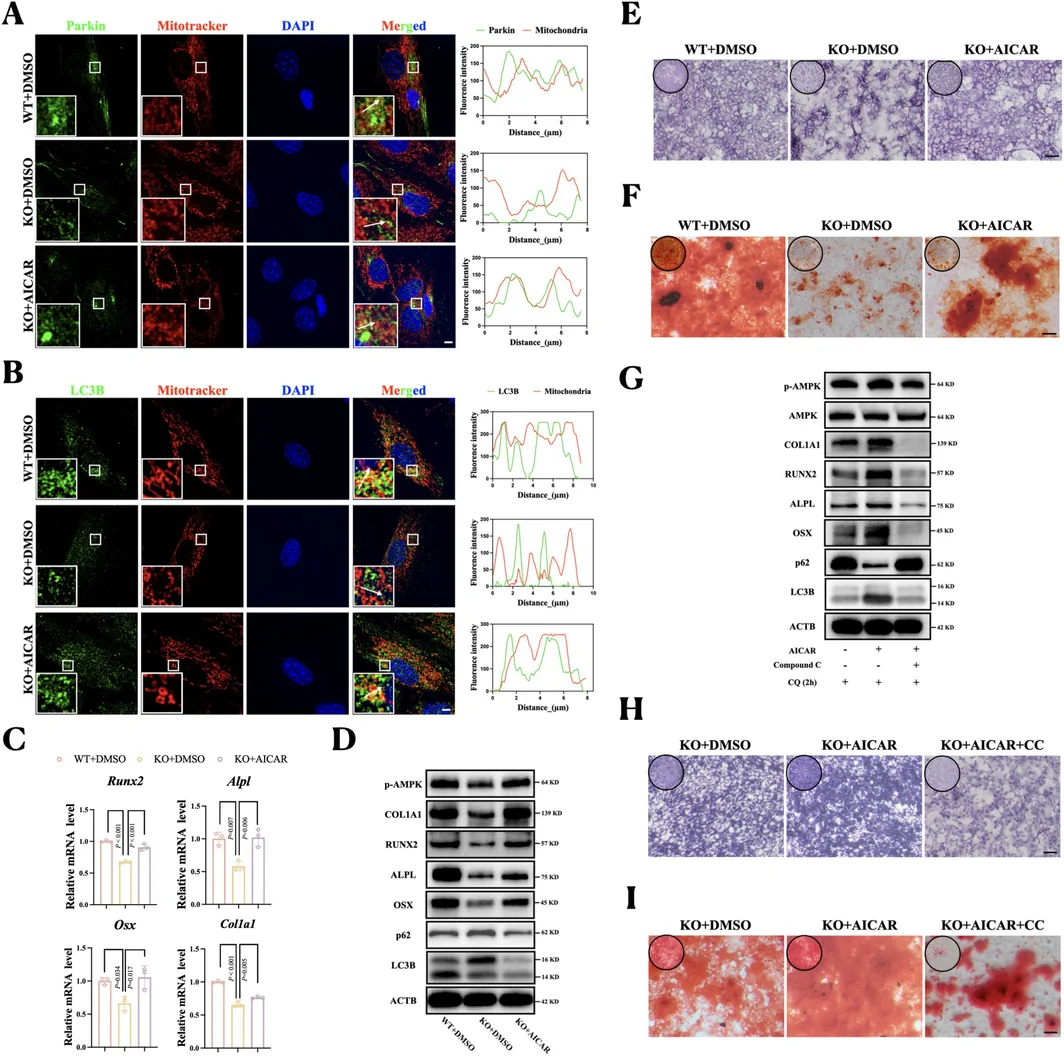
AMPK activation by AICAR rescues mitophagy and osteogenesis of *Aff4* KO OMSCs. (A, B) Representative IF images (left) and corresponding intensity profiles (right) showing colocalization of mitochondria with Parkin or LC3B in OMSCs in the presence or absence of AICAR treatment (100 μM, 2 h). Mitochondria, red; Parkin, green; LC3B, green; Nuclei, blue. Intensity profiles denote fluorescent signals along the indicated lines. Scale bar: 5 μm. (C–F) OMSCs were cultured in osteogenic medium with or without AICAR (100 μM) for indicated durations. (C) qRT‐PCR assay assessing mRNA expression levels of *Runx2*, *Osx*, *Alpl* and *Col1a1* after 5 days. (D) WB analysis measuring protein levels of p‐AMPK, COL1A1, RUNX2, ALPL, OSX, p62 and LC3B after 5 days. (E) ALP staining after 7 days. Scale bar: 200 μm. (F) ARS staining after 14 days. Scale bar: 200 μm. (G–I) *Aff4*‐KO OMSCs were cultured in osteogenic medium with DMSO (control), AICAR (100 μM), or AICAR (100 μM) plus Compound C (10 μM), as indicated. (G) WB analysis showing protein levels of p‐AMPK, AMPK, COL1A1, RUNX2, ALPL, OSX, p62 and LC3B after 5 days. Chloroquine (CQ, 40 μM, 2 h). (H) ALP staining after 7 days. Scale bar: 200 μm. (I) ARS staining after 14 days. Scale bar: 200 μm.

In summary, AMPK signalling inhibition contributes to defective mitophagy and osteogenic differentiation of *Aff4*‐KO OMSCs.

## 4 Disccusion

Treatment of maxillofacial bone defects is challenging due to the concurrent functional and aesthetic needs. Osteogenic capacity of OMSCs, which underpins bone repair in the maxillofacial region, remains mechanistically elusive. AFF4 is known to regulate osteogenic differentiation of diverse stem cell populations. Nevertheless, its role specifically within OMSCs for maxillofacial bone regeneration remains to be elucidated.

Here, the role of AFF4 in maxillofacial bone repair was first explored via a post‐extraction alveolar socket healing model. Significant upregulated AFF4 expression after tooth extraction hinted that AFF4 might be involved in alveolar socket healing. To test it, we further generated an *Aff4* knockout mouse model (*CAG‐iCre*; *Aff4* ^*fl/fl*^). Transgenic mouse line expressing Cre recombinase driven by the CAG promoter, which is both tamoxifen‐inducible and ubiquitously active, was used to circumvent the embryonic lethality of *Aff4* knockout mice [35, 36]. *Aff4* ^*fl/fl*^ littermates that underwent the same tamoxifen injection regimen served as WT controls, thereby excluding possible confounding influence of tamoxifen on bone metabolism [37]. In our study, extraction socket healing in WT mice was nearly complete by Day 21, consistent with the physiological healing process [38, 39], further indicating that tamoxifen administration did not adversely affect bone repair. Our in vivo results showed that postnatal inducible global *Aff4* knockout led to reduced formation of new bone mass in extraction socket. Alveolar socket healing requires migration, proliferation and osteogenesis of OMSCs in the early stage, followed by osteoclast differentiation and bone remodelling [38]. Our further histomorphometric analysis demonstrated that, in the early healing stage, delayed socket healing upon *Aff4* knockout was attributed to defective bone formation, not abnormal bone resorption. It is worth noting that osteoclastic activity during socket healing does not peak until Day 14 [38]. Therefore, whether AFF4 influences osteoclastic activity in the later bone remodelling stage remains to be elucidated by evaluation at later time points. Our finding aligns with the report by Dutt et al., in which *aff4* loss in zebrafish led to inhibited skeletal development and bone mineralization [40]. Notably, *Aff4* ablation did not disrupt gross body size or skeletal dimensions of mice in our study. This discrepancy may be attributed to the timing of inducible knockout in our experiment, which was after the rapid growth stage of murine skeletal development [41]. This approach provides a stable bone phenotype baseline, allowing us to pinpoint the role of AFF4 in skeletal homeostasis. OMSCs isolated from the mandible of *Aff4* KO mice exhibited impaired osteogenic capability in vitro, corroborating the positive regulatory role of AFF4 in osteogenic fate determination of other stem cells [17, 18, 42].

AFF4 has been reported to regulate autophagic activity [43], while its impact on mitophagy remains poorly understood. Mitophagy selectively degrades damaged mitochondria to guarantee mitochondrial quality control and cellular homeostasis [44]. Mitophagy blockade will lead to the accumulation of damaged, ROS‐generating mitochondria [45]. In this study, mitophagy inhibition upon *Aff4* loss was validated at various mitophagy stages, spanning from initiation, progression and final degradation of the mitophagosome. Accordingly, mitochondrial dysfunction and oxidative stress in *Aff4*‐KO OMSCs were also observed.

Mitophagy affects osteogenic activity via manipulating fate and function of osteoblastic lineage cells. Under chronic inflammatory conditions, accumulation of damaged mitochondria due to mitophagy inhibition impairs osteogenesis of hPDLSCs [46]. Mitophagy is also reported to prevent apoptosis, promote proliferation and osteogenesis of osteoblasts [47, 48, 49]. Mitophagy ensures the normal bioenergetic function of mitochondria, thereby meeting heightened ATP requirements during physiological osteogenesis. Moreover, mitophagy is also found to deliver amorphous calcium phosphate accumulated in mitochondria of osteoblastic lineage cells into extracellular collagen matrix, thus contributing to biomineralization process [27]. However, another study proposed the negative correlation between mitophagy and osteogenic potential of osteoblasts in type 2 diabetes osteoporosis [50]. Divergent findings on the role of mitophagy in bone formation may arise from context‐dependent variations in basal mitophagy levels and functional preferences. Consequently, the net effect of mitophagy on osteogenesis should be interpreted on a context‐specific basis. In our study, *Aff4* knockout compromised osteogenesis and mitophagy, and downregulated AMPK expression in OMSCs. Given the established roles of AMPK in bone metabolism and mitophagy [24, 25], we employed the pharmacological modulators AICAR (agonist) and Compound C (inhibitor) to investigate whether AMPK signalling is involved in the compromised mitophagic and osteogenic capacity of *Aff4‐*KO OMSCs. Compound C treatment suppressed osteogenesis to an even greater extent than that observed in DMSO‐treated *Aff4*‐KO OMSCs. This effect may be partly attributable to the known off‐target inhibition of BMP signalling by Compound C [51]. Given this limitation, complementary genetic approaches, such as AMPK overexpression or siRNA‐mediated knockdown, will be required to unequivocally define the specific contribution of AMPK in future studies.

In conclusion, our study reveals that alveolar bone injury stimulates AFF4 expression, which then regulates osteogenic differentiation of OMSCs and alveolar bone repair. Dysfunctional AMPK‐mitophagy under the condition of AFF4 loss contributes to the compromised osteogenic activity of OMSCs (Figure 6). Overall, our investigation provides deeper insights into the function of AFF4 in bone biology and may offer clues for developing therapeutic strategies to promote maxillofacial bone regeneration.

**FIGURE 6:**
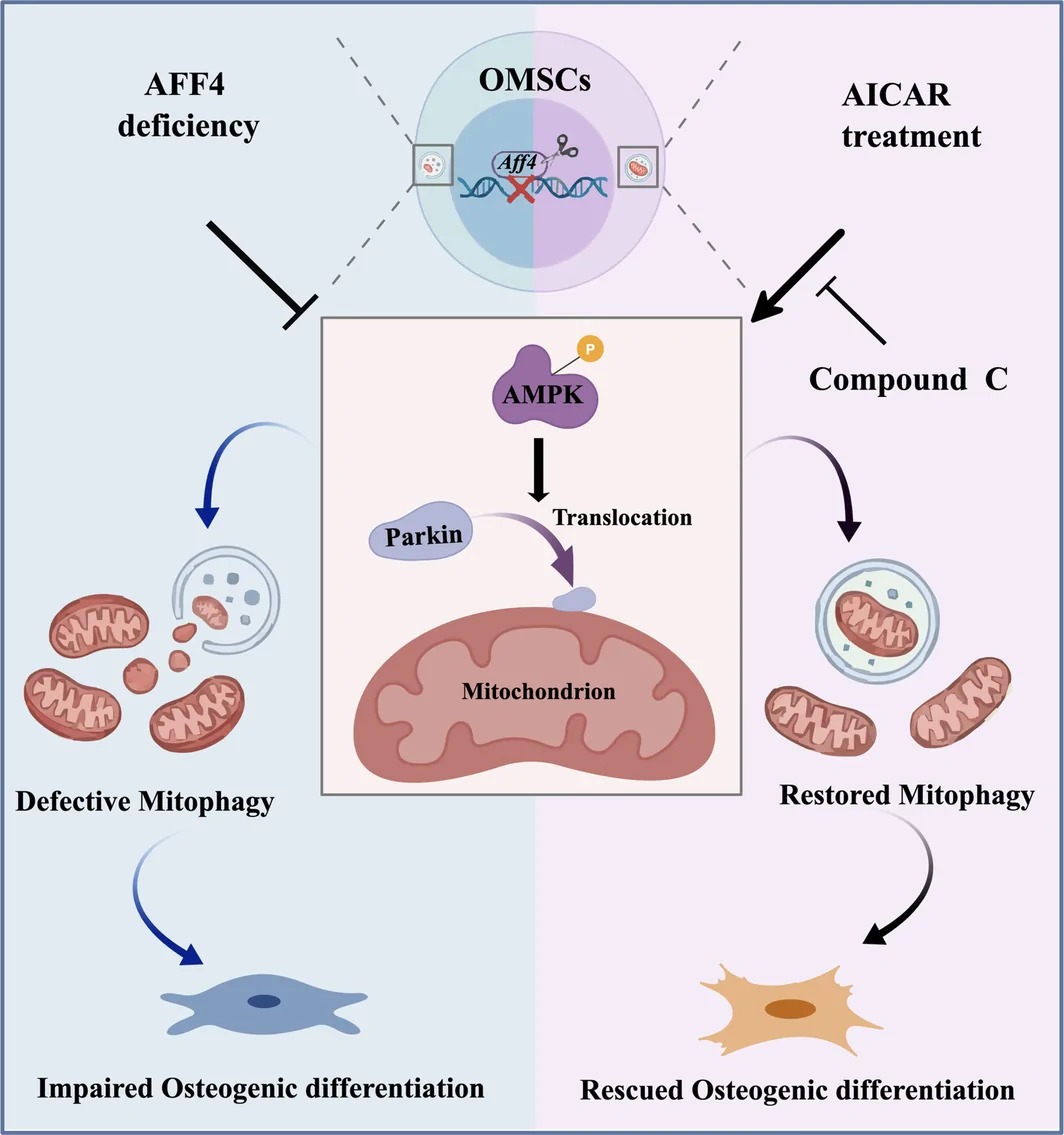
Schematic diagram showing molecular mechanisms underlying AFF4‐regulated osteogenesis of OMSCs (Figure created with BioGDP.com). In OMSCs, *Aff4* ablation inhibits AMPK signalling and downstream mitophagy, leading to impaired osteogenic differentiation. AICAR rescues the osteogenic potential of *Aff4*‐KO OMSCs in an AMPK‐dependent manner via mitophagy activation, as co‐treatment with Compound C effectively abrogated this effect.

Two major limitations of this study should be acknowledged. One is that global inducible *Aff4* knockout may perturb the bone microenvironment in which OMSCs reside, thereby disrupting the intricate interplay among stem cells, immune cells and local signalling molecules. Future studies using conditional knockout mouse models targeting the osteoblastic lineage (e.g., *Prrx1‐Cre*; *Aff4* ^*fl/fl*^ and *Osx‐Cre*; *Aff4* ^*fl/fl*^) would help to validate the specific contribution of OMSCs. Another limitation is that we did not further clarify the specific molecular mechanisms underlying AMPK regulation of mitophagy under AFF4‐deficient conditions. Beyond canonical Parkin‐dependent mitophagy, AMPK is found to inhibit NIX‐dependent mitophagy, which targets functioning mitochondria [22]. Future investigations are warranted to delineate whether and how diverse mitophagy pathways are involved in AFF4‐regulated AMPK signalling.

## Author Contributions

**Li Zhu:** conceptualization; investigation; data curation; writing – original draft. **Shuo Chen:** investigation. **Guowen Luo:** data curation. **Tingyue Zhang:** data curation. **Hengyi Lin:** formal analysis; interpretation of data. **Yiling Chen:** formal analysis; interpretation of data. **Chenchen Zhou:** conceptualization; writing – review and editing. **Shujuan Zou:** conceptualization; writing – review and editing.

## Funding

This research was supported by the National Natural Science Foundation of China (82271017, 32571424).

## Ethics Statement

All experimental procedures were reviewed and approved by the Ethics Committee of the West China College of Stomatology (WCHSIRB‐AT‐2025‐543).

## Conflicts of Interest

The authors declare no conflicts of interest.

## Supporting information


**Table S1:** Antibody.
**Table S2:** Primer sequences.
**Figure S1:** *Aff4* loss after pubertal growth peak bring negligible impact on gross growth. (A) WB analysis measuring AFF4 protein level in maxillae of WT and *Aff4*‐knockout (KO) mice. (B) Gross photographs of WT and *Aff4*‐KO mice at 4 weeks post‐induction. (C–D) Images of micro‐CT three‐dimensional reconstruction of maxilla and mandible of mouse in (B). (E) Sagittal sections of mandible in (D).
**Figure S2:** (A, B) IF staining showing MSCs surface markers, CD44 and CD99 expressed in OMSCs. (C) Representative confocal images showing mitochondrial morphology in WT and *Aff4* KO OMSCs. Scale bar: 5 μm. (D) Live cell imaging showing mitochondrial membrane potential in WT and *Aff4‐*KO OMSCs measured by JC‐1 assays. Scale bar: 5 μm. (E) Quantification of the ratio of red (JC‐1 aggregate) to green (JC‐1 monomer) fluorescence intensity in (D) (*n* = 4). (F) Representative MitoSOX staining showing mitochondrial superoxide in OMSCs. Scale bar: 5 μm. (G) Quantification of MitoSOX fluorescent signals in (F) (*n* = 3). (H) Representative confocal images showing intracellular reactive oxygen species (ROS) in OMSCs detected by of 2,7‐dichlorodihydrofluorescein diacetate (DCFH‐DA; green) staining. Scale bar: 5 μm. (I) Quantification of intracellular ROS levels in (H) (*n* = 5). (J) WB analysis showing protein levels of p‐DRP1, DRP1, FIS1, OPA1, MFN1 and MFN2 in WT and *Aff4‐*KO OMSCs.

## Data Availability

The data that support the findings of this study are available from the corresponding author upon reasonable request.
