# Supplementary material for: AFF4 Loss Compromises Osteogenesis of Orofacial Mesenchymal Stem Cells During Alveolar Bone Repair via AMPK‐Mediated Mitophagy
Source: Cell Prolif. 2026 Sep 29:e70284. Online ahead of print. doi: 10.1111/cpr.70284 (PMC13624416; doi:10.1111/cpr.70284)
Supplement: Supplementary file 1 [file CPR-9999-e70284-s001.docx]

**Supplementary Materials**

**Table S1. Antibody**

| **Antibody** | **Source and identifier** |
| --- | --- |
| Rabbit anti-AFF4 | 14662-1-AP; Proteintech |
| Rabbit anti-Collagen I | R26615; ZENBIO |
| Rabbit anti-RUNX2 | ET1612-47; Huabio |
| Rabbit anti-ALPL | ET1601-21; Huabio |
| Rabbit anti-OSX | HA722817; Huabio |
| Rabbit anti-AMPK | R380431; ZEN-BIO |
| Rabbit anti-phospho-AMPK Alpha (Thr172) | 80209-6-RR; Proteintech |
| Rabbit anti-LC3B | R382687; ZENBIO |
| Rabbit anti-p62 | R382862; ZENBIO |
| Rabbit anti-Parkin | HA722952; Huabio |
| Rabbit anti-PINK1 | PK05715; Abmart |
| Rabbit anti-TOMM20 | R382451; ZENBIO |
| Rabbit anti-FIS1 | 505821; ZENBIO |
| Rabbit anti-DRP1 | Abcam; ab184247 |
| Rabbit anti-DRP1 (Phospho Ser616) | YP1318; Immunoway |
| Rabbit anti-Mitofusin 1 | R381599; ZENBIO |
| Rabbit anti-Mitofusin 2 | R340604; ZENBIO |
| Rabbit anti-OPA1 | R382025; ZENBIO |
| HRP-conjugated Beta Actin Monoclonal antibody | HRP-60008; Proteintech |
| Goat anti-Rabbit IgG (H&L) HRP | L3012; Signalway Antibody |

**Table S2. Primer sequences**

| **Primer** | **Sequence (5’-3’)** |
| --- | --- |
| *Gapdh*_F(Mouse) | TGATGGGTGTGAACCACGAG |
| *Gapdh*_R(Mouse) | AGTGATGGCATGGACTGTGG |
| *Aff4*_F(Mouse) | ATGCCAAACCATAGCTGGAGA |
| *Aff4*_R(Mouse) | AGAGCACAGTGCATGCAAAC |
| *Runx2*_F(Mouse) | ATCCCCATCCATCCACTCCA |
| *Runx2*_R(Mouse) | GGGGTGTAGGTAAAGGTGGC |
| *Osx*_F(Mouse) | AGTGGTTTGGGGTTTGTTCCT |
| *Osx* _R(Mouse) | AGGCCAGCTCACTCTTGTTC |
| *Alpl_*F(Mouse) | CATGCCCAGTCCAGAGAAGAG |
| *Alpl*_R(Mouse) | CTCACAGCGTTCCTGGTTTG |
| *Col1a1*_F(Mouse) | GCAAGAGGCGAGAGAGGTTT |
| *Col1a1*_R(Mouse) | GACCACGGGCACCATCTTTA |
| *Ampkα1*_F(Mouse) | GGAAAGTGAAGGTGGGCAAG |
| *Ampkα1*_R(Mouse) | GGCTTTCCTTTTCGTCCAACC |
| *Ampkα2*_F(Mouse) | GCAAAGTGAAGACTACCAGGTG |
| *Ampkα2*_R(Mouse) | AGTAATCCACGGCAGACAGG |

**Figure S1**

**Figure S1. *Aff4* loss after pubertal growth peak bring negligible impact on gross growth.** (A) WB analysis measuring AFF4 protein level in maxillae of WT and *Aff4*-knockout (KO) mice. (B) Gross photographs of WT and *Aff4*-KO mice at 4 weeks post-induction. (C-D) Images of micro-CT three-dimensional reconstruction of maxilla and mandible of mouse in (B). (E) Sagittal sections of mandible in (D).

**Figure S2**

**Figure S2.** (A, B) IF staining showing MSCs surface markers, CD44 and CD99 expressed in OMSCs. (C) Representative confocal images showing mitochondrial morphology in WT and *Aff4* KO OMSCs. Scale bar: 5 μm. (D) Live cell imaging showing mitochondrial membrane potential in WT and *Aff4-*KO OMSCs measured by JC-1 assays. Scale bar: 5 μm. (E) Quantification of the ratio of red (JC-1 aggregate) to green (JC-1 monomer) fluorescence intensity in (D) (n=4). (F) Representative MitoSOX staining showing mitochondrial superoxide in OMSCs. Scale bar: 5 μm. (G) Quantification of MitoSOX fluorescent signals in (F) (n=3). (H) Representative confocal images showing intracellular reactive oxygen species (ROS) in OMSCs detected by of 2,7-dichlorodihydrofluorescein diacetate (DCFH-DA; green) staining. Scale bar: 5 μm. (I) Quantification of intracellular ROS levels in (H) (n=5). (J) WB analysis showing protein levels of p-DRP1, DRP1, FIS1, OPA1, MFN1, and MFN2 in WT and *Aff4-*KO OMSCs.
